# Evaluation of Pneumococcal Surface Protein A as a Vaccine Antigen against Secondary *Streptococcus pneumoniae* Challenge during Influenza A Infection

**DOI:** 10.3390/vaccines7040146

**Published:** 2019-10-11

**Authors:** Sean Roberts, Clare M. Williams, Sharon L. Salmon, Jesse L. Bonin, Dennis W. Metzger, Yoichi Furuya

**Affiliations:** Department of Immunology and Microbial Disease, Albany Medical College, Albany, NY 12208, USA; SER80@pitt.edu (S.R.); williac10@amc.edu (C.M.W.); SalmonS@amc.edu (S.L.S.); boninj@amc.edu (J.L.B.); metzged@amc.edu (D.W.M.)

**Keywords:** influenza-pneumococcal co-infection, pneumococcal vaccination, pneumococcal surface protein A, *Streptococcus pneumoniae*, Prevnar

## Abstract

Secondary bacterial pneumonia is responsible for significant morbidity and mortality during seasonal and pandemic influenza. Due to the unpredictability of influenza A virus evolution and the time-consuming process of manufacturing strain-specific influenza vaccines, recent efforts have been focused on developing anti-*Streptococcus pneumoniae* immunity to prevent influenza-related illness and death. Bacterial vaccination to prevent viral-bacterial synergistic interaction during co-infection is a promising concept that needs further investigation. Here, we show that immunization with pneumococcal surface protein A (PspA) fully protects mice against low-dose, but not high-dose, secondary bacterial challenge using a murine model of influenza A virus-*S. pneumoniae* co-infection. We further show that immunization with PspA is more broadly protective than the pneumococcal conjugate vaccine (Prevnar). These results demonstrate that PspA is a promising vaccine target that can provide protection against a physiologically relevant dose of *S. pneumoniae* following influenza infection.

## 1. Introduction

During seasonal and pandemic influenza, *S. pneumoniae* secondary bacterial infection is a key contributor to death. In fact, during the 1918 influenza pandemic, the presence of secondary pneumococci in the lungs is believed to have been one of the primary determinants of death [1]. Similarly, during the 2009 influenza pandemic, bacterial infection was a common cofactor in hospitalized patients [2,3]. We [4] and others [5,6,7] have successfully recapitulated the heightened susceptibility to secondary bacterial infections observed in the clinical setting using a mouse model of influenza-bacterial co-infection.

The Prevnar vaccine consists of capsular polysaccharides of the 7 or 13 most common serotypes of *S. pneumoniae* conjugated to a carrier protein. The Prevnar vaccine is recommended for children to prevent invasive pneumococcal diseases [8]. However, we have recently reported that Prevnar only partially protects against influenza–pneumococcal co-infection in a mouse model [9]. Similarly, vaccination with Pneumovax 23, a pneumococcal polysaccharide-based vaccine without a carrier protein, only protects about 39% to 41% of adults against secondary bacterial infection [10,11]. Recent efforts have been directed towards using pneumococcal surface protein A (PspA) as a vaccine candidate since it is highly conserved among the >90 serotypes [12,13]. PspA is classified into three families that consist of six clades [14]. Despite the variability in PspA among serotypes, immunization with recombinant PspA induces protective cross-reactive anti-PspA antibodies in mice [15] and humans [12,16,17]. Further, since the introduction of the Prevnar vaccination, serotype replacement to those not covered in the vaccine has been occurring. In contrast, PspA clade distribution has remained stable [12]. This indicates that newer immunization strategies consisting of several clades of PspA will likely provide heterologous and serotype-independent protection. Indeed, we [18] and others [19,20,21] have shown that vaccination with PspA provides protection against single pneumococcal challenge. In the current study, we assess the protective efficacy of PspA as a vaccine antigen against secondary *S. pneumoniae* infection following influenza A virus challenge.

## 2. Materials and Methods 

### 2.1. Anti-Pneumococcal Vaccination of Mice

Specific Pathogen Free, 8-week-old female C57BL/6Ncr mice were purchased from Charles River Laboratories (Wilmington, MA, USA). To induce anti-*Streptococcus pneumoniae* immunity, mice were vaccinated intramuscularly (i.m.) either with 3 μg of Pneumococcal surface protein A (family 1 Clade 2) plus 0.2 mg of aluminum hydroxide, 3 μg of Prevnar13 (Pfizer, New York, NY, USA), or PBS (Life Technologies, Carlsbad, CA, USA) given in a 100 μL volume. Mice were boosted 3 weeks post-prime and bled at week 4 for antibody quantification. Mice were housed within the Animal Research Facility of Albany Medical College. All experimental procedures were approved by the Institutional Animal Use and Care Committee (Protocol Number 17-03006). The following reagent was obtained through BEI Resources, NIAID, NIH: *Streptococcus pneumoniae* Family 1, Clade 2 Pneumococcal Surface Protein A (PspA UAB055) with C-Terminal Histidine Tag, Recombinant from *Escherichia coli*, NR-33178.

### 2.2. Antibody ELISA

Anti-pneumococcal antibody titers were quantified as previously described, with slight modifications [9]. In brief, sera from immunized mice were serially diluted and added to 96-well maxisorp plates (Thermo Fisher Scientific, Waltham, MA, USA) pre-coated with 2 μg of recombinant PspA or unconjugated PPS3 (ATCC, Manassas, VA, USA) for anti-PspA or anti-PPS3 antibody. For anti-A66.1 or anti-D39 antibody responses, plates were coated with 5 × 10^6^ CFU equivalents of heat-inactivated *S. pneumoniae* type 3 strain A66.1 or type 2 D39. Antigens were diluted in bicarbonate carbonate buffer (pH 9.5). To determine the titer, 50% of the maximal binding was used as the cut-off using log nonlinear regression (GraphPad Prism 6, La Jolla, CA, USA).

### 2.3. Influenza-S. pneumoniae Co-Infection Model

To model influenza-pneumococcal co-infection, mice anaesthetized with isoflurane were intranasally (i.n.) challenged with 10–15 PFU of H1N1 strain A/Puerto Rico/8/1934 (PR8) in a 50 μL inoculum two weeks post-vaccination, as previously reported [9]. Weight was monitored daily and once mice began to recover their weight i.e., day 8–10 post-influenza, they were challenged with 40 μL of 1.5 × 10^4^ CFU *S. pneumoniae* type 2 strain D39 or 5 × 10^2^, 5 × 10^3^, or 5 × 10^4^ CFU of *S. pneumoniae* type 3 strain A66.1 diluted in PBS. Bacterial inoculum was back-titrated on blood agar plates to confirm the actual challenge doses. A66.1 (family 1, clade 2) was originally obtained from David Briles (University of Alabama in Birmingham) [22]. D39 (family 1, clade 2) was a kind gift from Guangchun Bai (Albany Medical College) [23]. Bacteria were cultivated in Todd-Hewitt broth, resuspended in fresh broth with 15% glycerol, aliquoted, and stored at −80 °C. Frozen stock was thawed and serially diluted in PBS prior to infection.

### 2.4. Statistical Analyses

All results are expressed as individual mouse data ± SD. For comparison between two groups, Student’s *t*-test was used. Analysis of variance (ANOVA) was used for comparison between three or more groups and for experiments with multiple variables. Bonferroni or Tukey post-test was used for multiple comparisons. Survival curves were analyzed by Kaplan–Meier log-rank test. *p* values < 0.05 were considered significant.

## 3. Results

### 3.1. PspA Protein-Based Vaccination Generates Greater Anti-Pneumococcal IgG Antibody Levels Compared to Prevnar

**Figure 1:**
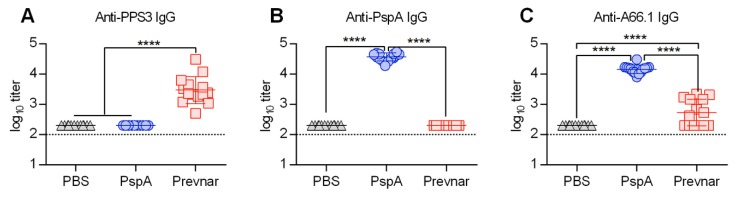
Pneumococcal surface protein A (PspA) vaccination induces robust anti-pneumococcal A66.1 antibody production. (**A**–**C**) PPS3- (**A**), PspA- (**B**) and A66.1 (**C**)-specific antibody titers in the serum day 7 post-vaccination. Mice (*n* = 12/group) were i.m. vaccinated with either PBS, PspA, or Prevnar and boosted on day 21. Sera were harvested on day 28 and analyzed by enzyme-linked immunosorbent assay (ELISA). Groups were compared using Analysis of variance (ANOVA) with Tukey’s post-test for multiple comparison. ****, *p* < 0.0001. Data shown are representative of two independent experiments.

Previously, we have shown that vaccination with Prevnar, the polysaccharide conjugate vaccine, only partially protects mice against secondary pneumococcal infection [9]. To determine if an *S. pneumoniae* protein-based vaccine can provide improved protection, we intramuscularly vaccinated mice with PspA. To quantify the antibody response following Prevnar and PspA vaccination, sera were harvested on day 7 post-boost and analyzed by antibody enzyme-linked immunosorbent assay (ELISA) (Figure 1A–C). Mice vaccinated with Prevnar or PspA had a 1.5 to 2 log increase in antigen specific IgG titer compared with PBS mice (Figure 1A,B). To directly compare PspA to the Prevnar vaccine response, ELISA plates were coated with heat-killed *S. pneumoniae* A66.1 serotype 3. The IgG response against the whole killed bacteria was significantly higher in PspA compared to Prevnar vaccinated mice (Figure 1C). 

### 3.2. Vaccine-Induced Protection against Co-Infection Is Bacterial Dose Dependent

**Figure 2:**
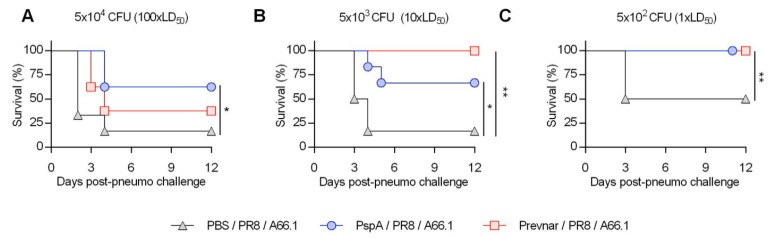
Survival of vaccinated mice following influenza-pneumococcus co-infection is bacterial dose dependent. (**A**–**C**) Mice were i.m. vaccinated with either PBS, PspA, or Prevnar13 and boosted on day 21 (*n* = 6/group). Two weeks post-vaccination mice were i.n. infected with 15 PFU of PR8 and i.n. challenged on day 9 post-PR8 with either 5 × 10^4^ (**A**), 5 × 10^3^ (**B**), 5 × 10^2^ CFU (**C**) of *S. pneumoniae* serotype 3 A66.1. * *p* < 0.01, ** *p* < 0.001; unvaccinated co-infected mice (PBS/PR8/A66.1) compared to either PspA or Prevnar vaccinated co-infected mice as determined by Log-rank test. High lethal (5 × 10^4^) dose survival data were repeated at least twice (**A**).

To determine if the enhanced anti-pneumococcal antibody response correlates with protection against secondary bacterial infection, vaccinated mice were challenged with a sub-lethal dose (10 PFU) of PR8 and co-infected with 5 × 10^4^ CFU (a dose equivalent to 100 × LD_50_ for co-infection) of *S. pneumoniae* A66.1 on day 9 post-influenza (Figure 2A). As expected, Prevnar-vaccinated (Prevnar/PR8/A66.1) mice were only partially protected, with 40% of the Prevnar vaccinated mice succumbing to the secondary infection within 3 days of the *S. pneumoniae* A66.1 challenge compared to 90% of unvaccinated co-infected mice (PBS/PR8/A66.1). Similarly, 66% of PspA vaccinated mice succumbed to co-infection. These results demonstrate that the PspA vaccination, like Prevnar, cannot fully overcome the defect in antibacterial protective mechanisms following influenza infection. The lack of protection was not due to a potential failure of vaccination, as both PspA and Prevnar vaccinated mice that were challenged with bacteria only were fully protected (Figure S1). In fact, vaccination protected 100% of mice challenged with a 20 × LD_50_ of single *S. pneumoniae* A66.1 infection (Figure S1).

Next, we infected mice with lower doses of bacteria to determine whether we had overwhelmed the protective efficacy of the vaccine (Figure 2B,C). Influenza-infected mice were challenged with 5 × 10^2^ bacterial CFU (1 × LD_50_ for co-infection) (Figure 2C). All the PspA and Prevnar vaccinated mice survived the 1 × LD_50_ secondary A66.1 challenge. In contrast, only 50% of unvaccinated co-infected mice survived (Figure 2C). No deaths were observed in the vaccinated or unvaccinated singly infected groups (Figure S1). Similar effects were observed in mice challenged with 5 × 10^3^ bacterial CFU (10 × LD_50_) (Figure 2B). These results suggest that vaccine-induced protection against co-infection is dependent on the bacterial challenge dose.

**Figure 3:**
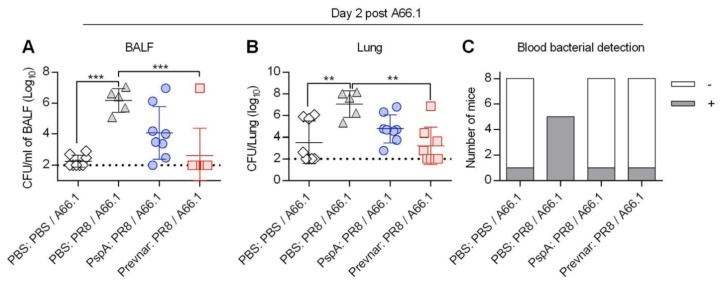
Reduced pulmonary and blood bacterial burden in PspA immunized mice following influenza-pneumo co-infection. (**A**–**C**) Mice were i.m. vaccinated with either PBS, PspA, or Prevnar and boosted on day 21 (*n* = 5–8/group). Two weeks post-vaccination mice were i.n. infected with 15 PFU of PR8 and i.n. challenged on day 10 post-PR8 with 5 × 10^3^ of *S. pneumoniae* serotype 3 A66.1. On day 2 post A66.1 infection, mice were sacrificed, and the bacterial CFU in the BALFs (**A**), lungs (**B**), and bloods (**C**) were enumerated. Statistical significance was determined by one-way ANOVA. **, *p* < 0.01, ***, *p* < 0.001. Lung burden data are representative of two independent experiments (**B**).

To assess whether the increases in survival were associated with enhanced bacterial clearance, bacterial burden in the bronchoalveolar lavage fluid (BALF), lung tissue, and blood was enumerated in mice immunized and challenged with 5 × 10^3^ CFU of *S. pneumoniae* A66.1 following influenza infection (Figure 3). As expected, unimmunized co-infected mice had significantly more bacteria in their BALF and lung tissue compared to unimmunized mice singly infected with bacteria. Both PspA and Prevnar immunized co-infected mice had fewer recoverable bacteria compared to the unimmunized co-infected mice (Figure 3A,B). Finally, all five unimmunized co-infected mice had bacteria in their blood, indicative of dissemination, compared to one out of eight mice in the bacteria-only and immunized groups (Figure 3C). These results suggest that immunization with PspA controls pulmonary bacterial outgrowth and bacteremia following a low-dose bacterial challenge in influenza-infected mice. 

### 3.3. PspA Immunization Is Protective against Serotype 2 Bacterial Challenge during Co-Infection

**Figure 4:**
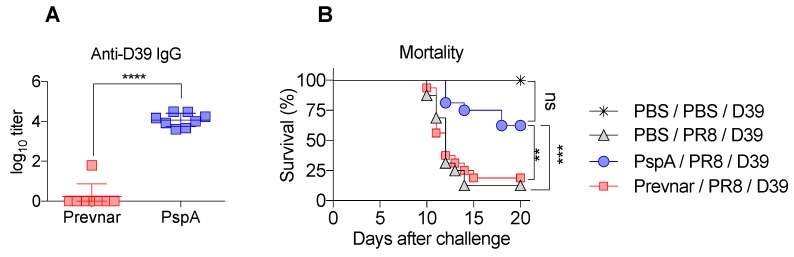
PspA vaccinated mice are protected against influenza-*S. pneumoniae* serotype 2 co-infection. Mice were i.m. vaccinated with either PBS, PspA, or Prevnar and boosted on day 21. (**A**) Sera were harvested on day 28 and analyzed by ELISA (*n* = 8 mice/group). (**B**) Two weeks post-vaccination mice were i.n. infected with 15 PFU of PR8 and i.n. challenged on day 9 post-PR8 with 1.5 × 10^4^ CFU *S. pneumoniae* type 2 strain D39 (*n* = 16/group). Survival data were combined from two independent experiments. Titer and survival data were analyzed by Student’s *t* test and Log-rank test, respectively. **, *p* < 0.01, ***, *p* < 0.001, ****, *p* < 0.0001.

To determine whether PspA vaccination can provide heterologous protection in a serotype independent manner during co-infection, we next used *S. pneumoniae* serotype 2 (strain D39), whose polysaccharide is not included in the Prevnar vaccine. We first measured anti-D39 antibody responses in mice immunized with PspA or Prevnar. As expected, mice immunized with Prevnar did not have detectable levels of anti-D39 antibody in contrast to PspA-immunized mice (Figure 4A). To see if this response was protective, we challenged these mice with a sublethal dose of D39 on day 9 post-influenza and monitored survival. PspA-immunized mice had significantly fewer deaths compared to unimmunized and Prevnar-immunized co-infected mice (Figure 4B). These results suggest that inclusion of PspA in the current Prevnar formulation would increase the breadth of protection. 

## 4. Discussion

During seasonal and pandemic influenza infection, commensal and/or opportunistic bacteria can invade the lower respiratory tract and cause pneumonia that can result in severe morbidity and/or death. In this study, we have shown that PspA vaccination can protect influenza-infected mice against secondary *S*. *pneumoniae* challenge. This protection was, however, dose-dependent; PspA vaccinated mice were fully protected against low-dose challenges, which were lethal for unvaccinated co-infected mice. This protection was strongly correlated with enhanced antibody production and bacterial clearance. 

This report extends our previous finding that Prevnar, a polysaccharide conjugate vaccine, only partially protects mice against co-infection [9]. Following that study, we hypothesized that the protection against co-infection was incomplete because polysaccharides have reduced immunogenicity compared to protein antigens and the response that is generated is not sufficient to overcome the influenza-induced defective antibacterial clearance mechanism(s). Our data now show that immunization with PspA, like polysaccharide-based vaccines, only partially protects influenza-infected mice against high doses of secondary bacterial pneumonia. These results support the idea that the suboptimal protection induced by vaccination is not a polysaccharide-specific phenomenon. Thus, a question remains as to why vaccine-induced anti-bacterial adaptive immunity cannot provide complete protection against post-influenza secondary *S. pneumoniae* infection. One of the dominating concepts in the influenza-bacterial co-infection field is centered on cytokine-mediated suppression of anti-bacterial immunity, including downregulation of phagocytic functions of innate immune cells [5,6,24,25,26,27,28,29,30,31]. Since PspA vaccination should not interfere with virus-induced cytokine responses, primary influenza infection is expected to impair anti-bacterial immunity in PspA vaccinated mice. Therefore, it is plausible that the observed suboptimal protection is due to an impaired opsonophagocytic killing of antibody-coated *S. pneumoniae*. This hypothesis is currently under investigation.

Several recent studies reported that PspA vaccination can induce a protective antibody response and enhance bacterial clearance during influenza-*S. pneumoniae* co-infection [21,32,33]. The distinctions between our studies and earlier works published by others are that (1) we compared our immunization to Prevnar13, the FDA approved anti-pneumococcal vaccine, and showed that PspA is equally as protective; (2) we showed that PspA can protect against more than one pneumococcal strain, specifically serotype 2 D39, which is a strain not included in Prevnar13; and (3) we showed that protection is dependent on the bacterial challenge dose. The dose escalation studies we conducted showed that 100% of mice immunized with PspA and subsequently challenged with 1 × LD_50_ of *S. pneumoniae* in a co-infection model survived. Under the same condition, only ~70% of PspA vaccinated mice survived the challenge of 100 × LD_50_. Nevertheless, our results agree with the other studies in that PspA vaccination can provide protection against secondary *S. pneumoniae* infection. How influenza A virus infection impairs anti-bacterial immunity is an area that is under active investigation. This is important because defective bacterial clearance is not due to insufficient antibody production or downregulation of Fcγ receptors needed to mediate antibody-dependent cellular phagocytosis [9].

It is important to note that PspA vaccination has been evaluated in phase 1 clinical trial and PspA was found to be safe and immunogenic—i.e., high levels of cross-clade antibodies to heterologous PspA molecules were detected in vaccinated individuals [17]. However, a theoretical issue was raised with the use of PspA as a vaccine antigen, due to a low sequence homology between PspA and human cardiac myosin [34]. Thus, there is a concern that PspA vaccination could lead to autoantibodies that may cause autoimmune conditions. However, such concerns can be alleviated by the absence of clinical evidence linking anti-PspA antibodies and cardiac injuries. Nonetheless, future efforts regarding the PspA vaccination approach should focus on PspA peptide-based vaccines that do not contain the homology with human cardiac myosin.

In this study, we show for the first time, by focusing primarily on survival, that vaccine-induced protection against influenza-*S. pneumoniae* co-infection is bacterial dose-dependent. We further show that the protective efficacy of PspA immunization is comparable to Prevnar13-induced protection against secondary serotype 3 pneumococcal (A66.1) challenge. However, PspA, unlike Prevnar, was protective against D39, a serotype not covered in Prevnar. This was not unexpected since, although three families of PspA exist, antibodies against PspA are cross-reactive within families and are cross-protective in a single *S. pneumoniae* infection model [14,16]. Thus, we conclude that there may be benefits to using PspA as a vaccine antigen over a polysaccharide-based vaccine or as an additional antigen in the current vaccine formulation. However, it needs to be stressed that our PspA vaccination approach was tested on adult mice. We believe that PspA vaccination would also provide some level of protection in susceptible individuals such as infants and older adults. Additional studies assessing the ability of PspA vaccination approach to provide protection against co-infection in other age groups would be of interest.

## Supplementary Materials

The following are available online at https://www.mdpi.com/2076-393X/7/4/146/s1. Figure S1: Survival of vaccinated mice following single *S. pneumoniae* infection.
