# Supplementary material for: Evaluation of Pneumococcal Surface Protein A as a Vaccine Antigen against Secondary Streptococcus pneumoniae Challenge during Influenza A Infection
Source: Vaccines (Basel). 2019 Oct 11;7(4):146. doi: 10.3390/vaccines7040146 (PMC6963301; doi:10.3390/vaccines7040146)
Supplement: Supplementary file 1 [file vaccines-07-00146-s001.pdf]

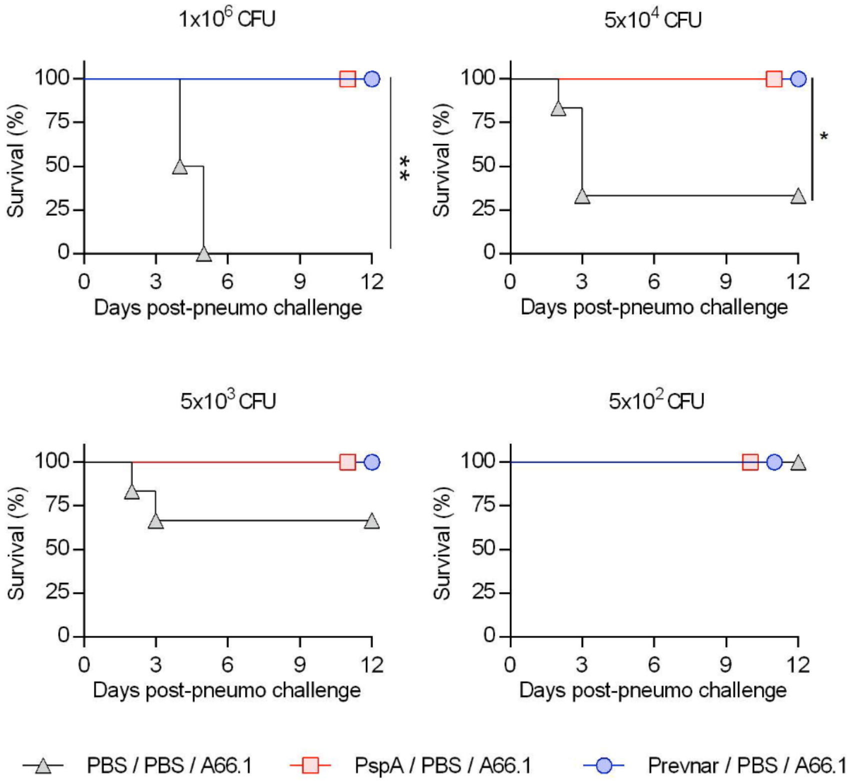

Figure S1. Survival of vaccinated mice following single *S. pneumoniae* infection. Mice were i.m. vaccinated with either PBS, PspA, or Prevnar and boosted on day 21 (n = ~6/group). Two weeks post-vaccination mice were i.n. infected with either 1×10^6, 5×10^4, 5×10^3, 5×10^2 CFU of *S. pneumoniae* serotype 3 A66.1 and survival was monitored for 12 days. *$p < 0.05$, **$p < 0.01$ as determined by Log-rank (Mantel-Cox) test.
